# Indigo Carmine: Between Necessity and Concern

**DOI:** 10.3390/jox13030033

**Published:** 2023-09-20

**Authors:** Madalina-Elena Ristea, Otilia Zarnescu

**Affiliations:** Faculty of Biology, University of Bucharest, Splaiul Independentei 91-95, R-050095 Bucharest, Romania; ristea.madalina-elena@s.bio.unibuc.ro

**Keywords:** indigo carmine, food dye, textile dye, diagnostic agent, toxicity

## Abstract

Dyes, such as indigo carmine, have become indispensable to modern life, being widely used in the food, textile, pharmaceutical, medicine, and cosmetic industry. Although indigo carmine is considered toxic and has many adverse effects, it is found in many foods, and the maximum permitted level is 500 mg/kg. Indigo carmine is one of the most used dyes in the textile industry, especially for dyeing denim, and it is also used in medicine due to its impressive applicability in diagnostic methods and surgical procedures, such as in gynecological and urological surgeries and microsurgery. It is reported that indigo carmine is toxic for humans and can cause various pathologies, such as hypertension, hypotension, skin irritations, or gastrointestinal disorders. In this review, we discuss the structure and properties of indigo carmine; its use in various industries and medicine; the adverse effects of its ingestion, injection, or skin contact; the effects on environmental pollution; and its toxicity testing. For this review, 147 studies were considered relevant. Most of the cited articles were those about environmental pollution with indigo carmine (51), uses of indigo carmine in medicine (45), and indigo carmine as a food additive (17).

## 1. Introduction

Dyes are organic compounds that are hydro or oil-soluble, natural or obtained through chemical synthesis [1,2]. For the past 158 years, dyes were obtained from natural sources, but the discovery of the first synthetic organic dye, mauveine, by William Perkin in 1865, revolutionized the dye industry and initiated the production of synthetic dyes globally [2,3,4].

Dyes are characterized by their capacity of absorbing light radiation in the visible spectrum (from 380 to 750 nm) and then reflecting the complementary colors. They are applied to the substrates and provide their permanent color that can resist fading upon exposure to different environmental factors, such as water, light, oxidizing agents, and microbial attack. Due to these advantages, dyes have been intensively used in various fields, such as the food, textile, medicine, pharmaceutical, cosmetic, plastic, rubber, and paper industry [2,5,6,7,8,9,10]. On the other hand, the extensive and intense uses of dyes produce impressive amounts of wastewater containing carcinogenic and toxic dyes that end up in the environment and *eventually* impact *human health* [5,6,7,8,9,10]. Thus, the dyes meet the needs of the population, but at the same time, they are responsible for ecological and sanitary changes in the water resources, soil, and atmosphere [11]. Nowadays, water pollution is one of the biggest challenges concerning the entire world, due to the large amount of dyes used in different industries [12]. Globally, it has been estimated that approximately 100,000 types of synthetic and natural dyes are produced, which represents between 7 × 108–1 × 109 kg/a year, but a significant amount is lost during the manufacturing processes [13]. Since dyes are intensively used to add colors to *different types of fabrics*, major sources of wastewater are textile industries that release non-biodegradable compounds into natural waters [14,15]. Also, to make products more attractive for consumers, dyes are present in processed foods, as well in pharmaceutical goods [16]. Furthermore, dyes have many uses in medical field, such as biological or vital stain, for histolopathology and tracing for perfect orientation of excised surgical specimens [17].

**Figure 1:**
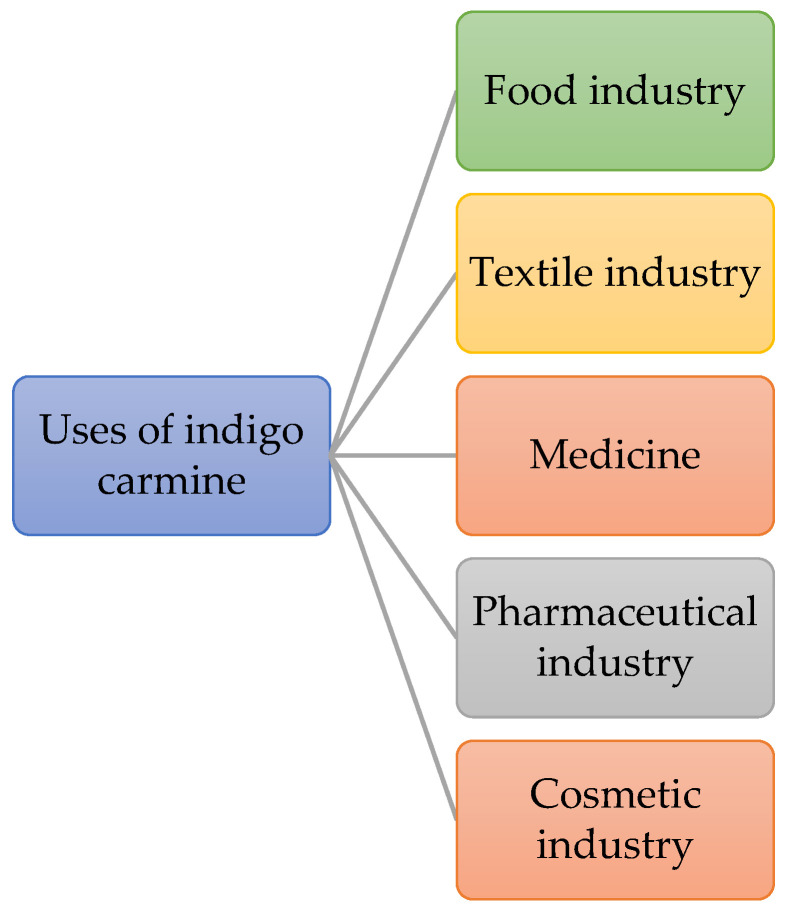
Uses of indigo carmine.

Despite its toxic potential, indigo carmine has a broad spectrum of uses in foods, textiles, medicine, pharmaceuticals, and cosmetics (Figure 1) [12,18,19,20,21,22]. So, indigo carmine is one of the very useful and necessary dyes used in many fields, but at the same time due to the potential adverse effects on the population and the environment, it raises many concerns.

In recent years, there have been many articles about indigo carmine in the literature. Most of them are about removing indigo carmine from wastewater, testing its toxicity, and its uses in medicine. Even if indigo carmine is a widely used dye, there are no reviews in the literature summarizing all its applications, or the adverse effects on humans and the environment and its toxicity. In this review article, we provide *an overview* on the *available data* regarding the structure and properties of indigo carmine, its manufacture, its uses and applications, and its adverse effects on human health and the environment.

## 2. Materials and Methods

To the best of our knowledge, no reviews on the uses and potential impact of exposure to indigo carmine have been published. An in-depth search of articles related to indigo carmine was performed using PubMed, Scopus, and Google Scholar databases. We used the following keywords: “indigo carmine” AND “Food”, “indigo carmine”, AND “textile”, “indigo carmine” AND “medicine”, “indigo carmine” AND “pollution”, “indigo carmine” AND “toxicity”.

A total of 147 studies were considered relevant by searching abstracts and titles. Of these, 111 studies were original articles, 23 were review articles, and 7 were book chapters. We included in our review 17 articles that mentioned indigo carmine as food additive, 5 articles about indigo carmine in the textile industry, 51 articles about environmental pollution with indigo carmine (including methods developed for the removal of indigo carmine from wastewater), 45 articles about uses of indigo carmine in medicine, and 13 toxicology studies (9 in vivo toxicology studies and 4 in vitro toxicology studies).

Additional data sources (EFSA/European Food Safety Authority, EINECS/European Inventory of Existing Commercial Chemical Substances; FAO/Food and Agriculture Organization of the United Nations, General Standard Food Additives, IUPAC/International Union of Pure and Applied Chemistry and JECFA/Joint FAO/WHO Expert Committee on Food Additives) were also searched.

## 3. The Structure and Properties of Indigo Carmine

**Figure 2:**
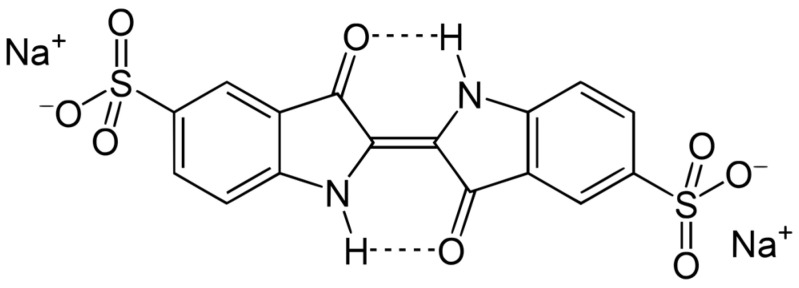
Chemical structure of indigo carmine.

Indigo carmine is a dark blue powder with the molecular formula C_16_H_8_N_2_Na_2_O_8_S_2_ and a molecular weight of 466.367 [23,24,25,26,27,28,29]. Its chemical structure presents two essential groups, NaSO_3_ and a chromophore group. NaSO_3_ provides the property of the dye to dissolve in water, and the chromophore group gives the compound its characteristic color. The chromophore group is a conjugate system of a C=C bond replaced by two C=O groups and two NH groups [27,30]. The chemical structure of the indigo carmine molecule is shown in Figure 2.

Indigo carmine has more than 100 synonyms. The most commonly used names are indigotine, indigotindisulfonate sodium, E132, CI natural blue II, FD&C Blue No.2, Acid blue 74, Brilliant blue, and CI (1975) No. 73015 [23,24,25,26,27,28,31]. According to the IUPAC (International Union of Pure and Applied Chemistry), indigo carmine is also called 3,3′-dioxo-2,2′-bis-indolyden-5,5-disulfonic acid disodium salt [23,25,26,32].

According to the European Commission Regulation 231/2012, Joint FAO/WHO Expert Committee on Food Additives, and U.S. Food and Drug Administration, indigo carmine consists of a mixture of disodium 3,3′-dioxo-2,2′bi-indo-lydidene-5,5′-disulphonate and disodium 3,3′-dioxo-2,2′-bi-indolylidene-5,7′-disulphonate, as well as auxiliary coloring substances and colorless components represented by sodium chloride and/or sodium sulfate. Thus, indigo carmine is often described as a sodium salt, but also as a calcium and potassium salt [25,31].

Indigo carmine must contain at least 85% total dyes calculated as the sodium salt, and disodium 3,3′-dioxo-2,2′-bi-indolylidene-5,7′-disulphonate not more than 18%. It also contains 0.2% water-insoluble substances, 1% other auxiliary coloring substances, 0.5% organic compounds, 0.01% unsulfonated primary aromatic amines, and 0.2% neutral ether-extractable substances [25,31].

Indigo carmine has poor pH stability, so after a week, it will appreciably fade if kept at a pH level of 3 to 5, will considerably fade at a pH 7, and will completely fade at a pH 8. Complete fading occurs in alkalis, such as 10% sodium carbonate and 10% sodium hydroxide. Also, color fading occurs in 10% of sugar systems. Indigo carmine has very poor light and oxidation stability and moderate heat stability, and is the least soluble one among food dyes, with a solubility of 1.6 g in 100 mL of water at 25 °C and slightly soluble in ethanol. Dissolved in water, indigo carmine gives the solution a blue color at neutrality, blue-violet in acid pH, and green to yellow-green in base pH. If it is dissolved in concentrated sulfuric acid, it yields a blue-violet solution that turns blue when diluted with water [25,33,34]. Indigo carmine has a good resistance to reducing agents, but it is very sensitive to oxidizing agents and has very poor compatibility with food components [34]. Table 1 shows the properties of indigo carmine.

## 4. The Manufacture of Indigo Carmine

Indigo is among the first known dyes and was originally obtained from the leaves of *Indigofera tinctoria*, *Indigofera suifruticosa*, or *Isatis tinctoria*, where it occurs as indican, an indoxyl glycoside. The enzyme indimulsin hydrolyzes the indican into indoxyl and glucose. By oxidizing, indoxyl is transformed into indigo. Since indigo carmine has been increasingly used since the 20th century, its extraction from plants has been replaced by its production through chemical synthesis.

Synthesis of indigo involves the reaction of aniline, formaldehyde, and hydrogen cyanide, affording phenylglycinonitrile that is then hydrolyzed to N-phenylglycine. To produce indoxyl, N-phenylglycine is treated with a molten mixture of sodamide, sodium, and sodium potassium hydroxide at 200 °C under ammonia pressure. The indoxyl undergoes further oxidative dimerization to form indigo. These processes produce large amounts of toxic waste products and need sophisticated purification processes [25,39,40].

Indigo carmine is obtained through sulfonation of indigo (2,2′-bindoline-3,3′-dione), which involves heating indigo in the presence of sulfuric acid, and it is subsequently subjected to purification processes [25,35].

## 5. Indigo Carmine in the Food Industry

The food industry represents one of the main fields in which indigo carmine is intensively used, being considered a food additive [16,27]. Food additives are widely used in developed countries to improve the taste, color, aroma, and texture and extend the shelf life of food or beverages [16,41,42]. In the food industry, synthetic dyes are preferred, because they present numerous advantages, such as stability in contact with light, oxygen, or another pH, as well as providing uniform color to food, excellent solubility, low risk of contamination with microorganisms, and low production costs [34,36,41,43,44]. Before the approval of a dye in the food industry, numerous toxicity tests are required, such as acute and chronic effects, carcinogenicity, mutagenicity, teratogenicity, toxicity on the reproductive system, and the degree of accumulation in the body, but also the identification of the LD 50 is performed [45,46].

Indigo carmine is heavily used in the food industry because the blue pigment is difficult to obtain from natural sources, and the dyes that are obtained do not have the same stability or coloring power and are not as affordable as synthetic dyes [34,44]. Thus, indigo carmine is part of the group III of synthetic food dyes used for a wide range of food products [35,42].

Indigo carmine is generally unstable in the presence of oxidizing or reducing agents, sugars, and salts because they alter the unsaturated conjugate group that gives it its characteristic color. Therefore, the recommended shelf life of indigo carmine in the food industry is 4 to 6 years [25].

The Codex Alimentarius Commission allows the use of indigo carmine in food and beverages with maximum permitted levels between 50 and 450 mg/kg, as mentioned in the General Standard Food Additives [47]. Also, the European Food Safety Authority has approved maximum levels for the use of indigo carmine in food and beverages ranging from 50 to 500 mg/kg [16]. The U.S. Food and Drug Administration (USFDA) has not decided maximum levels for this food additive, but it allows use under good manufacturing practice in a number of foods as established in the Code of Federal Regulations [48].

There are numerous analytical methods used for the identification and determination of indigo carmine within food. The most common used ones are thin-layer chromatography, high-performance liquid chromatography (HPLC) [49,50,51,52], ion chromatography, spectroscopy [16], voltammetry, differential pulse polarography, and capillary electrophoresis [51,53,54,55,56].

### Adverse Effects of Indigo Carmine Consumption

According to EFSA [25] and GSFA [47], indigo carmine is found within many food products (Table 2), although it presents a potential risk to human health [27,36]. The ingestion of indigo carmine can cause nausea, vomiting, diarrhea, and skin irritation [27,41].

The toxicity of indigo carmine was first evaluated by the JECFA (Joint FAO/WHO Expert Committee on Food Additives), which established a daily intake of 0–2.5 mg/kg body weight in 1969, increasing to 5 mg/kg body weight in 1975. The EFSA stated, based on available research, that indigo carmine does not show developmental toxicity, genotoxicity, or changes in hematological parameters in chronic toxicity tests [43,44]. Although the dye concentrations used are evaluated for consumer safety, their purity specifications allow certain concentrations of unsulfonated aromatic amines, which can reach 100 mg/kg of dye. If the average dye concentration is 500 mg/food kg, the amine concentration can reach 50 μg/food kg. If an amount of 15 mg/kg body weight of dye is ingested daily, the level of aromatic amines may be 1.5 μg/kg body weight. Thus, indigo carmine, and other synthetic blue dyes too, can affect human health through the indirect consumption of amines that have genotoxic and carcinogenic effects [44]. For indigo carmine, the level of unsulfonated aromatic amines has not yet been established. The European Food Safety Authority considered that the presence of unsulfonated aromatic amines, calculated as aniline, at the specifications limit of 0.01%, would not raise a concern [57].

Another concern is the interaction between food dyes and metal ions because it can change their stability, toxicity, and physico-chemical properties of both the dyes and the metal ions. Indigo carmine forms stable complexes with various ions, such as copper, zinc, cobalt, and nickel. It is essential to investigate the interference of the indigo carmine with metal ions since they are involved in almost all known important reactions and metabolic routes, and this dye is found in many food supplements and drugs [58].

## 6. Indigo Carmine in the Textile Industry

Dyes have been used in textile industry for more than 4000 years, but up the 19th century, they were obtained from natural sources, mostly plants, insects, and minerals [49]. Originally, indigo was obtained from the plants *Indigofera tinctoria* and *Isatis tentoria*, which were used to dye textiles in India, China, and Egypt [40,59]. Thus, the dyes were less toxic, less polluting, and produced fewer allergies compared to synthetic ones [3].

Indigo carmine has been identified in textile objects dating back to the 18th century. It was mainly used for dyeing silk, wool, and leather products [60], but also for dyeing polyester and denim fibers [6,61].

At the end of 19th century, with the development of chemical synthesis techniques, indigo became one of the most important and profitable products in the chemical industry [30]. In the 20th century, synthetic indigo still represents one of the most important products of the textile industry, almost completely replacing plant-derived indigo [40,62].

Among all dyes, indigo carmine remains one of the most used dyes in the textile industry [18,59,63,64], especially for dyeing denim. For instance, between 3 and 12 g of dye is needed to color a pair of jeans [40,59,61]. Thus, in the last decades, its production has increased, reaching up to 50,000 tons/years, with it being necessary to obtain it through chemical synthesis [64].

### Indigo Carmine and Water Pollution

The textile industry is considered a major source of pollution due to the discharge of non-biodegradable, acidic pH, and strongly oxidizable contaminants into natural water sources [4,26,59,61,65,66,67,68]. Dyes, including indigo carmine, cause extensive environmental pollution and pose a threat to aquatic organisms and public health [12,34,59,66,68,69,70]. The contamination of water with dyestuff can increase the turbidity, obstruct the sunlight penetration in water, increase the chemical oxygen demand, and affect the photosynthesis of plants and growth of bacteria [59,66]. Also, the dyes accumulate in aquatic organisms that can intoxicate animals and human through food cycle [66]. Further, industrial dyes, such as indigo carmine, are one of the dominant chemicals that make water non-potable [2] and change the color and smell of water, even at very low concentrations [71]. Therefore, several methods have been used to try to remove indigo carmine from the aquatic environment, such as adsorption [9,12,20,61,67,72,73], ultrafiltration [74,75], nanofiltration [76,77], reverse osmosis [76], electrocoagulation [68], electrodialysis [26], oxidation [71], micellar catalysis [78], photocatalytic degradation [79], photolysis [80], and bacterial treatment [81].

Adsorption is considered the most advantageous due to its ease of operation, high efficiency in a wide pH range, high performance, and the possibility of reusing the adsorbent by its regeneration [12,82]. Adsorption is a physical or chemical phenomenon, in which the pollutant is attached to the surface of the adsorbent material. *Numerous studies have been carried out* on the removal of indigo carmine from water, using various adsorbent materials such as activated carbon [83,84,85], various organic or inorganic matrices [78], magnetic composites [67], geopolymers [86], chitosan [87], β-cyclodextrin, chitin-chitosan [12], synthesized cationic hydrogels [61], ionic/nonionic polystyrene adsorbents [88], magnesium oxide, magnesium hydroxide, calcium oxide, and calcium hydroxide [89].

The removal of indigo carmine from wastewater also depends on the concentration of the dye and the pH [61]. Adsorbates must have certain expectations, such as adsorption capacity, effectiveness to a wide range of dyes, easiness of regeneration, and mechanical strength [12].

Relatively recently, certain forms of waste have begun to be used as adsorbent material to remove various dyes from waters and have been found to be very effective in removing indigo carmine from the aquatic environment. The most used wastes for water decontamination are sunflower stalks, corn cobs, sugarcane stalks, rice husks, wheat husks, clay, and paper [67,90,91,92,93].

Electrocoagulation is an electrochemical treatment process that uses electrodissolvable metal electrodes to clean polluted water with metal hydroxides and can remove even the smallest dye particles. Through this process, 82.55% of the dye was removed from a solution containing 20.01 mg/L indigo carmine, thus being an effective but also affordable method [68].

Also, to remove indigo carmine from waters, different advanced oxidation processes can be used, such as photocatalysis using TiO_2_ [30], ultrasonic assisten electrocatalysis on MnO_2_ using peroxydisulfate as the oxidant [94], and catalysis on hematite-derived nanocomposites using H_2_O_2_ as the oxidant [95]. MnO_2_ is considered to be an effective sonocatalyst and sonophotocatalyst for the complete removal of indigo carmine from water, including small amounts. The rate of degradation depends on substrate concentration, reaction volume, and ultrasound frequency, and it is facilitated by acidic pH. The use of sonocatalysis paired with sonophotolysis increases the efficiency of degradation and mineralization of indigo carmine, so that it can be removed from polluted waters [94].

Another method of removing indigo carmine from water is ozonation, which can also be associated with sonolysis and photocatalytic processes. The best results were obtained when ozone was used together with ultrasound because they favor the diffusion of gases in the reaction medium and the formation of oxidizing species, such as the hydroxyl radical and peroxydryl. Although these methods remove the dye from the waters, toxic compounds are subsequently produced, which further pollute the aquatic environment [71]. For instance, the use of TiO_2_ as a photocatalyst did not remove the total organic content of the water even if the water color disappeared. The use of peroxydisulfate as an oxidation agent implies the generation of sulfate as a by-product, which pollutes the aquatic environment [95].

## 7. Indigo Carmine in Medicine

Indigo carmine is one of the dyes used in medicine because it has an impressive applicability in terms of diagnostic methods and surgical procedures due to its intense color that allows for the visualization of structures to be analyzed [32,96]. Indigo carmine was used for the first time in medicine in 1904 [97] and is generally considered to be biologically inert [98,99,100] and relatively safe for human health due to its predominantly renal excretion [32]. However, adverse effects after the usage of indigo carmine have been reported in the literature [98,100].

Indigo carmine is mainly excreted by the kidneys [101,102]; it quickly reaches the urine, and due to the small size of the particles, it allows them to be easily filtered by the kidneys without tubular resorption [103,104]. It has been observed that indigo carmine does not affect renal function and is cleared from the circulation in the bladder within 5–7 min [103].

Indigo carmine is used in orthopedics and trauma surgery for staining cystic lesions at the knee and hip level (a 0.8% solution) or in herniated disc surgery (a 10 or 20% solution) [99,105]. It is also used for intradiscal visualization through the preferential staining of the degenerated nucleus pulposus [106]. Thus, lesions and degenerated and abnormal areas are colored and can be visualized during the intervention [99,106,107]. To visualize the ganglions with the arthroscope, a 1:10,000 indigo carmine solution is injected intralesionally into the wrist ganglion. Thus, the ganglion’s stalk and the cyst appear colored and are easy to analyze [108]. Indigo carmine is a very useful method for visualizing a fistula and for differentiating superficial from intra-articular infections [109].

Indigo carmine is one of the most common types of vital dyes used in microsurgery, being often used in vasography, vasovasostomy, vasoepididymostomy, and gynecological laparoscopy at a concentration of 25% [101].

Indigo carmine is used in gynecological and urological surgeries in combination with cystoscopy to evaluate urethral conditions and urine production during genitourinary surgeries [98,100,104,110,111,112]. It is administered intravenously, and the recommended dye concentration is 0.4% [104,112]. Indigo carmine is often used in gynecology because, unlike methylene blue, it has not been reported to induce teratogenic changes [113].

Imai et al. [106], described a new technique for visualization the dissectable layer in vaginal hysterectomy by staining it with vasopressin and indigo carmine. Indigo carmine delineates and widens the dissectible layer, and the organ injury is avoided [114].

Indigo carmine is also used in performing angiography to differentiate between tumor tissue and normal tissue in the case of ischemia of the lower extremities. Therefore, the use of indigo carmine in angiography provides essential visual information and is considered an important indicator in this pathology. The amount of indigo carmine used is 40 mg/5 mL without being diluted. The dye is injected through a catheter into the popliteal artery [115].

Indigo carmine is often used in chromoendoscopy for in vivo detection of pathologies of the colon mucosa, gastric mucosa, and urinary bladder [116,117]. It has been observed that the use of indigo carmine in chromoendoscopy can allow for the detection of gastric cancer in an early phase, but there are few clinical studies in the literature regarding its effectiveness in diagnosing the pathology. Generally, indigo carmine is considered a rapid diagnostic method and is used to distinguish between benign and malignant lesions, facilitates lesions analysis and delimits the borders of malignant lesions in the early stage, and estimates cancer invasion depth [21,37,118].

In addition, indigo carmine is useful in the recognition of intestinal lesions and in the diagnosis of patients infected with *Helicobacter pylori* [119]. Resindra et al. [120] highlighted that the use of indigo carmine in chromoendoscopy during stomach reconstruction interventions leads to a significant improvement in the completing the reconstruction result. It can also be used in chromoendoscopy to diagnose colorectal polyps [28] in the screening of neoplasia in patients with inflammatory bowel disease [28,121] and for detecting dysplasia in colitis [122].

Indigo carmine is used as a contrast agent during colonoscopy to differentiate between neoplastic and non-neoplastic colorectal lesions, being considered the most reliable method in diagnosing without performing a biopsy [123]. Frequently, indigo carmine is used to identify flat colorectal lesions. Normally a concentration of 0.5% is injected through the biopsy channel of endoscope, but the dye concentration can vary between 0.2 and 2% [124,125,126,127]. Also, another method for differentiating between neoplastic and non-neoplastic tissue is high pressure spraying with a solution of 0.035% indigo carmine [124].

Indigo carmine coats the mucosal structures through the accumulation of stains into the colonic pits, erosions, depressions, and ridges and allows for the immediate visualization of subtle changes and lesions. Thus, indigo carmine increased detection rates for flat and smaller lesions overlooked by conventional colonoscopy [126]. Neoplastic and non-neoplastic tissues can be differentiated based upon regular or irregular staining pit patterns and can subsequently guide targeted biopsies. Specifically, nonneoplastic tissue was defined by rounded or stellar pits, whereas neoplastic tissue was noted to have irregular, tubular, or villous pits [127]. The contrast stain is not absorbed into the mucosal epithelial cells, and because of this feature, magnifying chromoendoscopy with indigo carmine remains limited in accurately classifying advanced lesions, including invasive cancer and to predict the extent of submucosal invasion [128]. However, the sensitivity, specificity, and accuracy of magnifying chromoendoscopy with indigo carmine 0.2% are 96.0%, 72.2%, and 92.1%, respectively [129].

Indigo carmine can also be used in microendoscopy of the lacrimal glands, allowing for the identification of pathologies of the lacrimal mucosa [117].

### Adverse Effects of Using Indigo Carmine in Medicine

Although indigo carmine is generally considered a safe, biologically inactive dye, numerous adverse effects associated with its intravenous administration have been reported [98,117,130].

**Figure 3:**
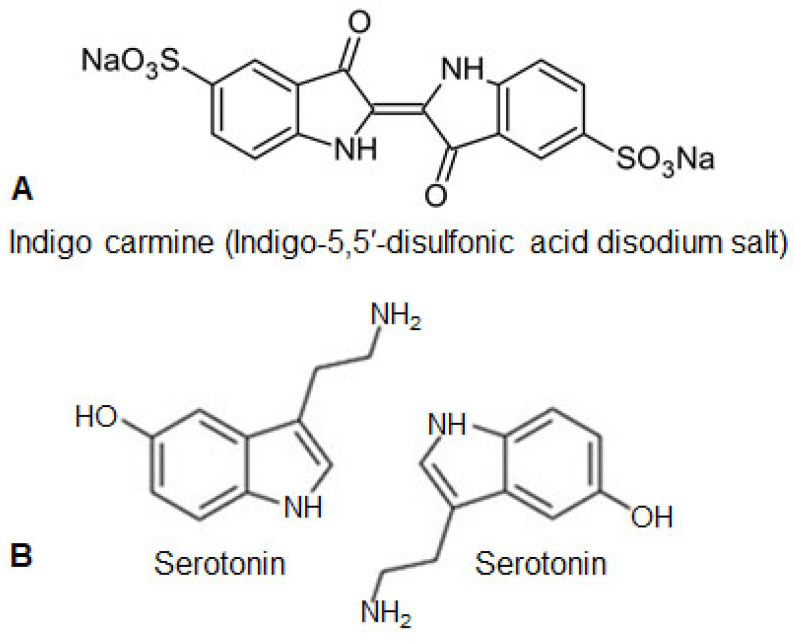
The structural formula of indigo carmine (**A**) and two molecules of serotonin arranged in a mirror image resembling indigo carmine (**B**).

Several studies have reported that indigo carmine has vasopressor properties [32,97,100,101,102,103,110,111,131,132,133] because its intravenous administration can cause transient stimulation of alfa-adrenergic receptors; can cause increases in diastolic and systolic blood pressure; and can reduce cardiac output, volume, and rate [32,110,130,132]. It can also cause reflex bradycardia with decreased cerebral vascular volume, as well as arrhythmias, but heart attacks have been rarely reported [100,103]. Craik et al. [132] observed that indigo carmine causes a statistically significant increase in both systolic and diastolic blood pressure with a maximum value recorded 5 min after administration. After 10 min, a significant decrease in heart rate was observed [97,100,132]. The increase in blood pressure is thought to be due to the molecular similarity of indigo carmine to serotonin because the molecular structure of indigo carmine resembles two molecules of 5-hydroxytryptamine (5-HT) arranged in a mirror image (Figure 3) [97,100,111,131,132,133]. However, cases of hypotension are very rare [43,100,111,133]. Indigo carmine has also been reported to affect blood oxygen saturation, but the decrease in oxygen levels is not very large [104].

Adverse effects of indigo carmine after skin contact have also been reported. After the use of indigo carmine in various surgeries, the dye extravasates into the skin and can cause severe irritation [27,103,134,135]. Fortunately, the only symptoms that patients have are discoloration and edema that last from 24 to 48 h [27,103,134,135].

Rodriguez-Ferreras and Ruiz-Salazar [28] presented the case of a 10-year-old patient who presented tooth discoloration following the administration of methylphenidate that also contains indigo carmine in concentrations of 30–40 mg per capsule. Isolated cases have also been reported in which the teeth of patients who ingested drugs containing indigo carmine became yellow due to the color change of the dye depending on the pH. In addition, other dental conditions have been reported following the administration of drugs containing indigo carmine, such as increased cavities, gingival hypertrophy and bleeding, toothache, gingivitis, periodontitis, and gingival erythema [28].

Also, eye contact may cause permanent damage to the cornea and conjunctiva [27,63]. After injecting a 0.4% dye solution into the lacrimal sac, indigo-carmine-stained tissue was observed to exhibit superficial atrophy, with diminished goblet cells and subepithelial fibrosis leading to fibrous change due to inflammation [117].

## 8. Other Applications of Indigo Carmine

Indigo carmine is used as a photometric detector, as a microscopic dye, and as a redox indicator in analytical chemistry [18,36,136]. Because indigo carmine exhibits antioxidant properties related to the ability to scavenge anionic superoxide or dioxygen, it is used for ozone detection. It is also a chelator of minerals such as cooper, zinc, and cobalt to the extent that they participate in the Fenton reaction, which can help avoid oxidative stress [32,58,137,138].

Also, in the 1960s, indigo carmine was used to measure dissolved oxygen, and thus it was a quality indicator of beer. This process involved adding indigo carmine to a beer sample and taking a colorimetric measurement [139]. In addition, indigo carmine is also considered an antibacterial agent, an additive to poultry feed, and a hair dye [27,67].

## 9. Toxicity Testing of Indigo Carmine

The effect of indigo carmine on mortality has been tested on several species.

Gaunt et al. [140] administered concentrations of 150, 450, and 1350 mg/kg of indigo carmine to *Sus domesticus* for 90 days, and no mortality percentage was recorded after the experiment. A decrease in hemoglobin level and red blood cell count was observed after 45 and 90 days in males given 1350 mg/kg/day.

Hooson et al. [141] reported that they did not observe an increase in mortality rate of mice after administration of indigo carmine in concentrations of 0.4, 0.8, and 1.6% for 80 weeks.

Also, other studies were carried out to see other effects of the dye. Growth inhibition was observed in rats after administration of 2 and 5% indigo carmine for 2 years, but no effects on viability or pathological changes were reported [142]. Singh et al. [143] have highlighted that after oral administration of the dye to mice and rats, it has no carcinogenic and mutagenic effects. On the other hand, Ozaki et al. [144] reported that indigo carmine exhibits mutagenic activity and inhibits the growth of Bacillus subtilis species.

Dixit and Goyal [145] evaluated the toxic potential of indigo carmine on the male reproductive organ of mice. The mice were divided into two experimental groups. One group ingested 17 mg/kg/day of indigo carmine, and the other group ingested 39 mg/kg/day of dye. The experiment lasted 42 days. A significant increase in body weight and a significant reduction in tubular diameter, as well a decrease in sperm motility, were observed in both experimental groups. Also, a significant reduction in sperm density was observed in the group that received a higher dose of dye.

In the case of *Eisenia andrei*, the lethal dose 50 could not be established because the mortality rate was low even in the case of the highest concentration (166.67 mg/cm^2^) after 48 h of exposure. The researchers estimated that after 72 h of intoxication, the concentration that can induce the lethal effect of 50% of individuals is 75.70 mg/cm^2^, considering that the toxic effect of indigo carmine is dependent on the exposure time [27].

Although the dye has antioxidant properties, it was observed that following the evaluation of the effects of indigo carmine treatment on the liver of rats subjected to ischemic and reperfusion injuries, indigo carmine is not sufficient to mitigate hepatocyte damage. In addition, the dye acts synergistically with ischemic lesions, thus favoring the destruction of hepatocyte membranes [32].

Mahadevan et al. [113] compared the effects of indigo carmine with those of methylene blue on granulosa luteal cell function in vitro. Methylene blue was found to significantly reduce the cells production of progesterone, while indigo carmine had no effect.

Indigo carmine was tested in vitro on cell cultures prepared from bovine coccygeal intervertebral disc tissue, and it was demonstrated that the staining efficacy and cytotoxicity were proportional to dye concentration, but significant toxicity was observed at concentrations higher than 2.5 mg/mL [146]. Furthermore, the effect of indigo carmine was tested in vitro on human chondrocytes, where a significant decrease in the number of viable cells was observed at concentrations of 10% and 100% [105] and on human fibroblasts where it was observed a reduction in cell proliferation at concentrations of 100, 250, 500, 1000, and 2000 μg/mL [21].

Yoo et al. [147] tested a dye mixture of indigo carmine and lipiodol on porcine models. They concluded that the dye mixture had reliable stability and visibility for localizing lung lesions on porcine models, but for safety, it is recommended to test the dye mixture on the lung surface within 8 h.

## 10. Conclusions

The use of dyes in various industries, especially in the food and textile industries, is a consequence of modern life. All dyes present many advantages, but at the same time, they can also be considered a cause of concern for the health of the population and the environment.

Since indigo carmine has many uses, but also numerous adverse effects, its use is a necessity, but also a concern. Although there are studies highlighting the adverse effects of indigo carmine consumption and the disastrous environmental effects resulting from its use in the textile industry, this dye is still intensively produced and used. For instance, is reported that after ingestion, there are adverse effects, such as nausea, vomiting, diarrhea, and skin irritation, but it is still found in many foods, including foods intended for children. In the future, more tests must be conducted on the adverse effects following the ingestion of indigo carmine. Later, depending on the results, other maximum permitted levels for consumption should be established.

Another concern is water pollution with indigo carmine. Because this dye is intensively used in the textile industry, it often ends up in water. For this reason, there is a growing research interest in the removal of indigo carmine from wastewater. Thus, more studies are needed to determine the concentrations of indigo carmine in wastewater and to find the most effective and affordable methods of removing the dye from wastewater. Also, in the future, there is a need to regulate the use of indigo carmine in the textile industry to minimize pollution.

Although indigo carmine has impressive applicability in diagnostic methods and surgical procedures, there are many studies in the literature reporting adverse effects arising from its use, such as hypertension, skin irritation, and edema.

*Moreover, nothing is known about the mechanism of uptake*, accumulation, transport, and cellular metabolism of indigo carmine.

In conclusion, indigo carmine is very useful and necessary in many fields, but due to potential adverse effects on humans and the environment, more experiments are needed to highlight the cellular and molecular aspects of the interaction of indigo carmine with biological structures in both aquatic and terrestrial environments.

## Figures and Tables

**Table 1:** Properties of indigo carmine dye.

Properties of Indigo Carmine Dye		Authors
Molecular formula	C_16_H_8_N_2_Na_2_O_8_S_2_	[26,27,35]
IUPAC name	3,3′-dioxo-2,2′-bis indolyden-5,5-disulfonic acid disodium salt	
EINECS	212-728-8	[25]
Consistency	powder	[26,27,35,36]
Color	dark blue	
Color index number	73,015	[25]
Molecular weight	466.367 g/mol	[23,26,37]
Melting point	>300 °C	[26]
Maximum absorption	608–612 nm	
Composition	dye content ~ 85%; ˂18% disodium 3,3′-dioxo-2,2′-bi-indolylidene-5,7′-disulphonate	
Reactive group	amines, phosphines, pyridines, salts basic, ketones, hydrocarbons, aliphatic unsaturated	
Purity	water-insoluble matter ˂ 2%; auxiliary coloring substances ˂ 1%; organic compounds (isatin-5-sulfonic acid, 5-sulfoanthranilic acid, anthranilic acid) ˂ 1%; unsulfonated primary aromatic amines ˂ 0.01%; ether extractable matter ˂ 0.2%; arsenic ˂ 3 mg/kg; lead ˂ 2 mg/kg; cadmium ˂ 1 mg/kg; mercury ˂ 1 mg/kg	[25,38]
Solubility	10 g/L	[27]
Density	0.4–0.6	

IUPAC = International Union of Pure and Applied Chemistry; EINECS = European Inventory of Existing Commercial Chemical Substances.

**Table 2:** Maximum permitted levels (MPL) of indigo carmine (E 132) in food according to the Annex II to Regulation (EC) No. 1333/2008 and GSFA [25,47].

Food Category No.	Food Category	Restriction/Exceptions	EFSA MPL mg/kg	GSFA MPL mg/kg
01.1.2	Dairy-based drinks, flavored and/or fermented		-	300
01.4	Flavored fermented milk products including heat-treated products		150	-
01.6.1	Unripened cheese	For use in surface treatment only	150	200
01.6.2.2	Rind of ripened cheese	Refers to the rind only of the cheese	-	100
01.6.3	Other creams		150	-
01.6.4.2	Flavored processed cheese, including containing fruit, vegetables, meat, etc.		-	100
01.6.5	Cheese analogues		-	200
01.7	Dairy-based desserts-pudding, fruit, or flavored yoghurt		-	150
01.7.3	Edible cheese rind		*quantum satis*	-
01.7.6	Cheese products	Only flavored unripened products	100	-
02.1.3	Lard, tallow, fish oil and other animal fats		-	300
02.3	Fat emulsions mainly of type oil-in-water, including mixed and/or flavored products based on fat emulsions		-	300
02.4	Fat-based desserts excluding dairy-based dessert		-	150
03.0	Edible ices, including sherbet and sorbet		150	150
04.1.2.11	Fruit fillings for pastries		-	150
04.1.2.5	Jams, jellies, marmalades		-	300
04.1.2.6	Fruit-based spreads		-	300
04.1.2.7	Candied fruit		-	200
04.1.2.8	Fruit preparations, including pulp, purees, fruit toppings and coconut milk		-	150
04.1.2.9	Fruit-based desserts, including fruit-flavored water-based desserts		-	150
04.2.2.3	Vegetables (mushrooms and fungi, roots and tubers, pulses and legumes, and aloe vera), and seaweeds in vinegar, oil, brine, or soybean sauce		-	150
04.2.2.7	Fermented vegetable (mushrooms and fungi, roots and tubers, pulses and legumes, and aloe vera) and seaweed products		-	300
04.2.4.1	Fruit and vegetable preparations excluding compote	Only *mostarda di frutta*	200	-
05.2	Confectionery including hard and soft candy, nougats		200–300	300
05.3	Chewing gum		300	300
05.4	Decorations for fine bakery wares, toppings (non-fruit) and sweet sauces	Only decorations, coatings, and sauces, except fillings	300–500	300
05.1.4	Cocoa and chocolate products	For use in surface decoration only	-	450
05.1.5	Imitation chocolate, chocolate substitute products		-	300
06.5	Cereal- and starch-based desserts (rice pudding, tapioca pudding)		-	150
06.6	Batters	Only for coating	500	-
07.2	Fine bakery wares (sweet, salty, savory) and mixes		200	200
08.2.3	Casings and coatings and decorations for meat	Only decorations and coatings except edible external coating of *pasturmas*	500	-
09.1.1	Fresh fish	For use in decoration, stamping, or branding the product only	-	300
09.2	Processed fish and fishery products including mollusks and crustaceans	Only surimi and similar products and salmon substitutes	500	-
09.2.1	Frozen fish, fish fillets, and fish products, including mollusks, crustaceans, and echinoderms	For use in surimi and fish roe products only	-	300
09.2.4.1	Cooked fish and fish products	For use in surimi and fish roe products only	-	300
09.2.4.2	Cooked mollusks, crustaceans, and echinoderms	For use in glaze, coatings, or decorations	-	250
09.3	Fish roe	Except sturgeon’s eggs (caviar)	300	-
09.3.3	Salmon substitutes, caviar and other fish roe products		-	300
09.3.4	Semi-preserved fish and fish products, including mollusks, crustaceans, and echinoderms, excluding products of food categories 09.3.1–09.3.3		-	300
09.4	Fully preserved, including canned or fermented fish and fish products, including mollusks, crustaceans, and echinoderms	Fish roe, sardines, surimi	-	300
10.1	Fresh eggs	For use in decoration, stamping, or branding the product only	-	300
10.4	Egg-based desserts (e.g., custard)		-	300
11.4	Other sugars and syrups (e.g., xylose, maple syrup, sugar toppings)		-	300
12.2.2	Seasonings and condiments	Only seasonings, for example, curry powder, tandoori	500	300
12.4	Mustards		300	300
12.5	Soups and broths		300	50
12.6	Sauces and similar products	Including pickles, relishes, chutney, and piccalilli; excluding tomato-based sauces	500	300
12.9	Protein products	Only meat and fish analogues based on vegetable proteins	100	-
13.2	Dietary foods for special medical purposes defined in Directive 1999/21/EC		50	-
13.3	Dietetic foods intended for special medical purposes (excluding products of food category 13.2)		50	50
13.4	Dietetic formulas for slimming purposes and weight reduction		-	50
13.5	Dietetic foods (e.g., supplementary foods for dietary use) excluding products of food categories 13.1–13.4 and 13.6		-	300
13.6	Food supplements		-	300
14.1.4	Water-based flavored drinks, including sport, energy, or electrolyte drinks and particulated drinks		100	100
14.2.2	Cider and perry	Excluding *cidre bouché*	200	200
14.2.4	Wines (other than grape)		200	200
14.2.6	Distilled spirituous beverages containing more than 15% alcohol		200	300
14.2.7	Aromatized alcoholic beverages (e.g., beer, wine and spirituous cooler-type beverages, low-alcoholic refreshers)		200	200
14.2.7.1	Aromatized wines	Except *americano*, *bitter vino*	200	-
14.2.7.2	Aromatized wine-based drinks		200	-
14.2.7.3	Aromatized wine-product cocktails		200	-
14.2.8	Other alcoholic drinks including		200	-
15.1	Snacks—potato-, cereal-, flour-, or starch-based (from roots and tubers, pulses, and legumes)		100	200
15.2	Processed nuts, including coated nuts and nut mixtures (with e.g., dried fruit)	Only savory-coated nuts	100	100
16	Desserts excluding products covered in categories 01, 03, and 04		150	-
17.1	Food supplements supplied in a solid form (capsules and tablets and similar forms, excluding chewable forms)		300	-
17.2	Food supplements supplied in a liquid form		100	-
17.3	Food supplements supplied in a syrup-type or chewable form		100–300	-

GFSA: General Standard on Food Additives; EFSA: European Food Safety; Authority; MPL: maximum permitted level.

## Data Availability

Not applicable.

## References

[1] Ngaha M.C.D., Njanja E., Doungmo G., Kamdem A.T., Tonle I.L. (2019). Indigo carmine and 2,6 dichlorophenolindophenol removal using cetyltrimethylammonium bromide-modified palm oil fiber: Adsorption isotherms and mass transfer kinetics. Int. J. Biomater..

[2] Khan I., Saeed K., Zekker I., Zhang B., Hendi A.A., Ahmad A., Ahmad S., Zada N., Ahmad H., Shah L.A. (2022). Review on methylene blue: Its properties, uses, toxicity and photodegradation. Water.

[3] Benkhaya S., Mrabet S., Elharfi A. (2020). A review on classifications, recent synthesis and applications of textile dyes. Inorg. Chem. Commun..

[4] Tkaczyk A., Mitrowska K., Posyniak A. (2020). Synthetic organic dyes as contaminants of the aquatic environment and their implications for ecosystems: A review. Sci. Total Environ..

[5] Ahmad A., Mohd-Setapar S.H., Chuong C.S., Khatoon A., Wani W.A., Kumar R., Rafatullah M. (2015). Recent advances in new generation dye removal technologies: Novel search for approaches to reprocess wastewater. RSC Adv..

[6] Kesraoui A., Selmi T., Seffen M., Brouers F. (2017). Influence of alternating current on the adsorption of indigo carmine. Environ. Sci. Pollut. Res. Int..

[7] Alencar L.V.T.D., Passos L.M.S., Soares C.M.F., Lima A.S., Souza R.L. (2020). Efficiency method for methylene blue recovery using aqueous two-phase systems based on cholinium-ionic liquids. J. Fashion Technol. Text..

[8] Pandey S., Do J.Y., Kim J., Kang M. (2020). Fast and highly efficient removal of dye from aqueous solution using natural locust bean gum based hydrogels as adsorbent. Int. J. Biol. Macromol..

[9] Ahmad M., Rehman W., Khan M.M., Qureshi M.T., Gul A., Haq S., Ullah R., Rab A., Menaa F. (2020). Phytogenic fabrication of ZnO and gold decorated ZnO nanoparticles for photocatalytic degradation of Rhodamine B. J. Environ. Chem. Eng..

[10] Khan S., Naushad M., Govarthanan M., Iqbal J., Alfadul S.M. (2022). Emerging contaminants of high concern for the environment: Current trends and future. Environ. Res..

[11] Ventura-Camargo B.C., Marin-Morales M.A. (2013). Azo dyes: Characterization and toxicity—A review. Text. Light Ind. Sci. Technol..

[12] Kekes T., Tzia C. (2020). Adsorption of indigo carmine on functional chitosan and β-cyclodextrin/chitosan beads: Equilibrium, kinetics and mechanism studies. J. Environ. Manag..

[13] Bouras H.D., Isik Z., Arikan E.B., Yeddou A., Bouras N., Chergui A., Favier L., Amrane A., Dizge N. (2020). Biosorption characteristics of methylene blue by two fungal biomasses. Int. J. Environ. Stud..

[14] Castillo-Suárez L.A., Sierra-Sánchez A.G., Linares-Hernádes I., Martínez-Miranda V., Teutli-Sequeira E.A. (2023). A critical review of textile industry wastewater: Green technologies for the removal of indigo dyes. Int. J. Environ. Sci. Technol..

[15] Fong W.M., Affam A.C., Chung W.C. (2020). Synthesis of Ag/Fe/CAC for colour and COD removal from methylene blue dye wastewater. Int. J. Environ. Sci. Technol..

[16] Pagnacco M., Maksimović J.P., Nikolić N.T., Bogdanović D.V.B., Kragović M.M., Stojmenović M.D., Blagojević S.N., Senćanski J.V. (2022). Indigo carmine in a food dye: Spectroscopic characterization and determining its micro-concentration through the clock reaction. Molecules.

[17] Wainwright M., Clark M. (2011). Dyes for the medical industry. Handbook of Textile and Industrial Dyeing: Applications of Dyes.

[18] Lakshmi U.R., Srivastava V.C., Mall I.D., Lataye D.H. (2009). Rice husk ash as an effective adsorbent: Evaluation of adsorptive characteristics for indigo carmine dye. J. Environ. Manag..

[19] Edwin D.S.S., Manjunatha J.G., Raril C., Girish T., Ravishankar D.K., Arpitha H.J. (2021). Electrochemical analysis of indigo carmine using polyarginine modified carbon paste electrode. J. Electrochem. Sci. Eng..

[20] El-Kammah M., Elkhatib E., Gouveia S., Cameselle C., Aboukila E. (2022). Enhanced removal of indigo carmine dye from textile effluent using green cost-efficient nanomaterial: Adsorption, kinetics, thermodynamics and mechanisms. Sustain. Chem. Pharm..

[21] Pasdaran A., Azarpira N., Heidari R., Nourinejad S., Zare M., Hamedi A. (2022). Effects of some cosmetic dyes and pigments on the proliferation of human foreskin fibroblasts and cellular oxidative stress; potential cytotoxicity of chlorophyllin and indigo carmine on fibroblasts. J. Cosmet. Dermatol..

[22] Tabti S., Benchettara A., Smaili F., Benchettara A., Berrabah S.E. (2022). Electrodeposition of lead dioxide on Fe electrode application to the degradation of indigo carmine dye. J. Appl. Electrochem..

[23] Kim I.S., Kim K.H., Shin S.W., Kim T.K., Kim J.I. (2005). Indigo carmine for the selective endoscopic intervertebral nuclectomy. J. Korean Med. Sci..

[24] Kim K.H., Kim Y.S., Kuh S.U., Park H.S., Park J.Y., Chin D.K., Kim K.S., Cho Y.E. (2013). Time and dose dependent cytotoxicities of ioxitalamate and indigocarmine in human nucleus pulposus cells. Spine J..

[25] EFSA ANS Panel (European Food Safety Authority Panel on Food Additives and Nutrient Sources Added to Food) (2014). Scientific opinion on the re-evaluation of indigo carmine (E 132) as a food additive. EFSA J..

[26] Caprarescu S., Miron A.R., Purcar V., Radu A.L., Sarbu A., Ion-Ebrasu D., Atanase L.I., Ghiurea M. (2016). Efficient removal of indigo carmine dye by a separation process. Water Sci. Technol..

[27] Pereira P.C.G., Reimao R.V., Pavesi T., Saggioro E.M., Moreira J.C., Correira F.V. (2017). Lethal and sub-lethal evaluation if indigo carmine dye and byproducts after TiO2 photocatalysis in the immune system of *Eisenia andrei* earthworms. Ecotoxicol. Environ. Saf..

[28] Rodriguez-Ferreras A., Ruiz-Salazar J. (2019). Indigo carmine related tooth discoloration. Excipients: A pending subject. Farm. Hosp..

[29] Secula M.S., Cretescu I., Petrescu S. (2011). An experimental study of indigo carmine removal from aqueous solution by electrocoagulation. Desalination.

[30] Vautier M., Guillard C., Herrmann J.M. (2001). Photocatalytic degradation of dyes in water: Case study of indigo and of indigo carmine. J. Catal..

[31] JECFA (Joint FAO/WHO Expert Committee on Food Additives) (2018). Compendium of Food Additive Specifications—86th Meeting.

[32] Rancan E.A., Frota E.I., de Freitas T.M.N., Jordani M.C., Évora P.R.B., Castro-E-Silva O. (2020). Evaluation of indigo carmine on hepatic ischemia and reperfusion injury. Acta Cir. Bras..

[33] JECFA (Joint FAO/WHO Expert Committee on Food Additives) (2010). Combined Compendium of Food Additive Specifications—All Specifications Monographs from the 1st to the 73rd Meeting (1956–2010).

[34] Olas B., Bialecki J., Urbańska K., Brys M. (2021). The effects of natural and synthetic blue dyes on human health: A review of current knowledge and therapeutic perspectives. Adv. Nutr..

[35] König J., Scotter M.J. (2015). Food colour additives of synthetic origin. Colour Additives for Foods and Beverages.

[36] Arvand M., Saberi M., Ardaki M.S., Mohammadi A. (2017). Mediated electrochemical method for the determination of indigo carmine levels in food products. Talanta.

[37] Nagao S., Tsuji Y., Sakaguchi Y., Takahashi Y., Minatsuki C., Niimi K., Yamashita H., Yamamichi N., Seto Y., Tada T. (2020). Highly accurate artificial intelligence systems to predict the invasion depth of gastric cancer: Efficacy of conventional white-light imaging, nonmagnifying narrow-band imaging, and indigo carmine dye contrast imaging. Gastrointest. Endosc..

[38] European Commission (2012). Commission regulation (EU) No 231/2012 of 9 March 2012 Laying Down Specifications for Food Additives Listed in Annexes II and III to Regulation (EC), No 1333/2008 of the European Parliament and of the Council. OJEU..

[39] Rebelo S.L.H., Linhares M., Simoes M.M.Q., Silva A.M.S., Neves M.G.P., Cavaleiro J.A.S., Freire C. (2014). Indigo dye production by enzymatic mimicking based on an iron (III) porphyrin. J. Catal..

[40] Steingruber E., Elvers B. (2004). Indigo and indigo colorants. Ullmann’s Encyclopedia of Industrial Chemistry.

[41] Okafor S.N., Obonga W., Ezeokonkwo M.A. (2016). Assessment of the health implications of synthetic and natural food colourants—A critical review. J. Pharm. Biosci..

[42] Martynov V.O., Brygadyrenko V.V. (2018). The influence of the synthetic food colourings tartrazine, allura red and indigo carmine on the body weight of *Tenebrio molitor* (Coleoptera, Tenebrionidae) larvae. Regul. Mech..

[43] Amchova P., Kotolova H., Ruda-Kucerova J. (2015). Health safety issues of synthetic food colorants. Regul. Toxicol. Pharmacol..

[44] Neves M.I.L., Silva E.K., Meireles M.A. (2021). Natural blue food colorants: Consumer acceptance, current alternatives, trends, challenges, and future strategies. Trends Food Sci. Technol..

[45] Merinas-Amo R., Martínez-Jurado M., Jurado-Güeto S., Alonso-Moraga A., Merinas-Amo T. (2019). Biological effects of food coloring in in vivo and in vitro model systems. Foods.

[46] Ahmed M.A., Al-Khalifa A.S., Al-Nouri D.M., El-din M.F.S. (2021). Dietary intake of artificial food color additives containing food products by school-going children. Saudi J. Biol. Sci..

[47] GSFA (General Standard Food Additives) Food Additive Details: GSFA Provisions for Indigotine (Indigo Carmine). https://www.fao.org/gsfaonline/additives/details.html?id=96&d-3586470-s=2&d-3586470-o=2&print=true.

[48] JECFA (Joint FAO/WHO Expert Committee on Food Additives) (2020). Safety Evaluation of Certain Food Additives: Prepared by the Eighty-Sixth Meeting of the JECFA.

[49] Kiseleva M.G., Pimenova V.V., Eller K.I. (2003). Optimization of conditions for the HPLC determination of synthetic dyes in food. J. Anal. Chem..

[50] Minioti K.S., Sakellariou C.F., Thomaidis N.S. (2007). Determination of 13 synthetic food colorants in water-soluble foods by reversed-phase high-performance liquid chromatography coupled with diode-array detector. Anal. Chim. Acta.

[51] Chao J., Feng F., Chen Z., Chu X. (2011). Highly sensitive determination of 10 dyes in food with complex matrices using SPE followed by UPLC-DAD-tandem mass spectrometry. J. Liq. Chromatogr. Relat..

[52] Feng F., Zhao Y., Yong W., Jiang G., Chu X. (2011). Highly sensitive and accurate screening of 40 dyes in soft drinks by liquid chromatography-electrospray tandem mass spectrometry. J. Chromatogr. B Analyt. Technol. Biomed. Life Sci..

[53] Huang H., Shih Y.C., Chen Y.C. (2002). Determining eight colorants in milk beverages by capillary electrophoresis. J. Chromatogr. A..

[54] Prado M.A., Boas L.F.V., Bronze M.R., Godoy H.T. (2006). Validation of methodology for simultaneous determination of synthetic dyes in alcoholic beverages by capillary electrophoresis. J. Chromatogr. A.

[55] European Commission (2013). Analysis of needs in post-market monitoring of food additives and preparatory work for future projects in this field. EFSA Support. Publ..

[56] Harp B.P., Miranda-Bermudez E., Barrows J.N. (2013). Determination of seven certified color additives in food products using liquid chromatography. J. Agric. Food Chem..

[57] EFSA ANS Panel (European Food Safety Authority Panel on Food Additives and Nutrient Sources Added to Food) (2023). Follow-up of the re-evaluation of indigo carmine (E 132) as a food additive. EFSA J..

[58] Zanoni T.B., Cardoso A.A., Zanoni M.V.B., Ferreira A.A.P. (2010). Exploratory study on sequestration of some essential metals by indigo carmine food dye. Braz. J. Pharm. Sci..

[59] Chowdhury M.F., Khandaker S., Sarker F., Islam A., Rahman M.T., Awual M.R. (2020). Current treatment technologies and mechanisms for removal of indigo carmine dyes from wastewater: A review. J. Mol. Liq..

[60] de Keijzer M., van Bommel M.R., Hofmann-de-Keijzer R., Knaller R., Oberhumer E. (2013). Indigo carmine: Understanding a problematic blue dye. Stud. Conserv..

[61] Sari M.M. (2010). Removal of acidic indigo carmine textile dye from aqueous solutions using radiation induced cationic hydrogels. Water Sci. Technol..

[62] Fabara A.N., Fraaije M.W. (2020). Production of indigo through the use of a dual-function substrate and a bifunctional fusion enzyme. Enzyme Microb. Technol..

[63] Othman I., Mohamed R.M., Ibrahim I.A., Mohamed M.M. (2006). Synthesis and modification of ZSM-5 with manganese and lanthanum and their effects on decolorization of indigo carmine dye. Appl. Catal..

[64] Pattanaik L., Duraivadivel P., Hariprasad P., Naik S.N. (2020). Utilization and re-use of solid and liquid waste generated from the natural indigo dye production process-a zero waste approach. Bioresour. Technol..

[65] dos Anjos F.S., Vieira E.F., Cestari A.R. (2002). Interaction of indigo carmine dye with chitosan evaluated by adsorption and thermochemical data. J. Colloid Interface Sci..

[66] Babu A.N., Reddy D.S., Sharma P., Kumar G.S., Ravindhranath K., Mohan G.V.K. (2019). Removal of hazardous indigo carmine dye from wastet water using treated red mud. Mater. Today Proc..

[67] Achieng G.O., Kowenje C.O., Lalah J.O., Ojwach S.O. (2021). Synthesis and characterization of FSB@Fe_3_O_4_ composites and application in removal of indigo carmine dye from industrial wastewaters. Environ. Sci. Pollut. Res. Int..

[68] Tanyol M., Yildirim N.C., Alparslan D. (2021). Electrocoagulation induced treatment of indigo carmine textile dye in an aqueous medium: The effect of process variables on efficiency evaluated using biochemical response of *Gammarus pulex*. Environ. Sci. Pollut. Res..

[69] Hessel C., Allegre C., Maisseu M., Charbit F., Moulin P. (2007). Guidelines and legislation for dye house effluents. J. Environ. Manag..

[70] Berradi M., Hsissou R., Khudhair M., Assouag M., Cherkaoui O., El Bachiri A., El Harfi A. (2019). Textile finishing dyes and their impact on aquatic environs. Heliyon.

[71] Ortiz E., Gόmez-Chávez V., Cortés-Romero C.M., Solís H., Ruiz-Ramos R., Loera-Serna S. (2016). Degradation of indigo carmine using advanced oxidation processes: Synergy effects and toxicological study. J. Environ. Prot. Sci..

[72] Gupta T., Ansari K., Lataye D., Kadu M., Khan M.A., Mubarak N.M., Garg R., Karri R.R. (2022). Adsorption of indigo carmine dye by *Acacia nilotica* sawdust activated carbon in fixed bed column. Sci. Rep..

[73] Ahmed M.A., Ahmed M.A., Mohamed A.A. (2023). Synthesis, characterization and application of chitosan/grapheme oxide/copper ferrite nanocomposite for the adsorptive removal of anionic and cationic dyes from wastewater. RSC Adv..

[74] Forgacs E., Cserháti T., Oros G. (2004). Removal of synthetic dyes from wastewaters: A review. Environ. Int..

[75] Karthik V., Saravanan K., Bharathi P., Dharanya V., Meiaraj C. (2014). An overview of treatments for the removal of textile dyes. J. Chem. Pharm. Sci..

[76] Abid M.F., Zablouk M.A., Abid-Alameer A.M. (2012). Experimental study of dye removal from industrial wastewater by membrane technologies of reverse osmosis and nanofiltration. Iran J. Environ. Health Sci. Eng..

[77] Liang C.Z., Sun S.P., Li F.Y., Ong Y.K., Chung T.S. (2014). Treatment of highly concentrated wastewater containing multiple synthetic dyes by a combined process of coagulation/flocculation and nanofiltration. J. Membr. Sci..

[78] Răducan A., Puiu M., Oancea P., Colbea C., Velea A., Dinu B., Mihăilescu A.M., Galaon T. (2019). Fast decolourization of indigo carmine and crystal violet in aqueous environments through micellar catalysis. Sep. Purif. Technol..

[79] Huy B.T., Paeng D.S., Thao C.T.B., Phuong N.T.K., Lee Y. (2020). ZnO-Bi_2_O_3_/graphitic carbon nitride photocatalytic system with H2O2-assisted enhanced degradation of indigo carmine under visible light. Arab. J. Chem..

[80] Zukawa T., Sasaki Y., Kurosawa T., Kamiko N. (2019). Photolysis of indigo carmine solution by planar vacuum-ultraviolet (147 nm) light source. Chemosphere.

[81] Li H.X., Xu B., Tang L., Zhang J.H.M., Mao Z.G. (2015). Reductive decolorization of indigo carmine dye with Bacillus sp. MZS10. Int. Biodeterior. Biodegrad..

[82] Wu L., Galanakis C.M. (2021). Analysis of food additives. Innovative Food Analysis.

[83] Gutiérrez-Segura E., Solache-Ríos M., Colín-Cruz A. (2009). Sorption of indigo carmine by a Fe-zeolitic tuff and carbonaceous material from pyrolyzed sewage sludge. J. Hazard. Mater..

[84] Zhang J., Zhang P., Zhang S., Zhou Q. (2014). Comparative study on the adsorption of tartrazine and indigo carmine onto maize cob carbon. Sep. Sci. Technol..

[85] Zhou Y., Lu J., Zhou Y., Liu Y. (2019). Recent advances for dyes removal using novel adsorbents: A review. Environ. Pollut..

[86] Siyal A.A., Shamsuddin M.R., Khan M.I., Rabat N.E., Zulfiqar M., Man Z., Siame J., Azizli K.A. (2018). A review on geopolymers as emerging materials for the adsorption of heavy metals and dyes. J. Environ. Manag..

[87] Cestari A.R., Vieira E.F.S., Tavares A.M.G., Bruns R.E. (2007). The removal of the indigo carmine dye from aqueous solutions using cross-linked chitosan—Evaluation of adsorption thermodynamics using a full factorial design. J. Hazard. Mater..

[88] Pan J., Zhou L., Chen H., Liu X., Hong C., Chen D., Pan B. (2021). Mechanistically understanding adsorption of methyl orange, indigo carmine, and methylene blue onto ionic/nonionic polystyrene adsorbents. J. Hazard. Mater..

[89] Ramesh T.N., Sreenivasa V.P. (2015). Removal of indigo carmine dye from aqueous solution using magnesium hydroxide as an adsorbent. J. Mater..

[90] Robinson T., Chandran B., Nigam P. (2002). Removal of dyes from a synthetic textile dye effluent by biosorption on apple pomace and wheat straw. Water Res..

[91] Ansari R., Seyghali B., Mohammad-khah A., Zanjanchi M.A. (2012). Application of nano surfactant modified biosorbent as an efficient adsorbent for dye removal. Sep. Sci. Technol..

[92] Shikuku V.O., Atina G.O.A., Kowenje C., Wani K.A., Jangid N.K. (2019). Removal of dyes from wastewater by adsorption onto low-cost adsorbents. Impact of Textile Dyes on Public Health and the Environment.

[93] Ahmad M.B., Soomro U., Muqeet M., Ahmed Z. (2020). Adsorption of indigo carmine dye onto the surface-modified adsorbent prepared from municipal waste and simulation using deep neural network. J. Hazard. Mater..

[94] Lekshmi K.P.V., Yesodharan S., Yesodharan E.P. (2018). MnO_2_ efficiently removes indigo carmine dyes from polluted water. Heliyon.

[95] Pavel O.D., Stamate A.E., Zavoianu R., Cruceanu A., Tirsoaga A., Birjega R., Brezestean I.A., Ciorita A., Culita D.A., Dias A.P.S. (2023). Mo-LDH-GO hybrid catalysts for indigo carmine advanced oxidation. Materials.

[96] Zaidan F., Freitas P.A.M. (2015). Flow injection analysis of indigo carmine using green coconut *(Cocos Nucifera* L.) fiber as a bioadsorbent. Int. J. Eng. Res..

[97] Erickson J.C., Widmer B.A. (1968). The vasopressor effect of indigo carmine. Anesthesiology.

[98] Naitoh J., Fox B.M. (1994). Severe hypotension, bronchospasm, and urticaria from intravenous indigo carmine. Urology.

[99] Park H.J., Lee S.M., Choi J.A., Park N.H., Kim H.S., Park S.I. (2010). Preoperative localization of cystic lesions in the knee using ultrasound-guided injection of indigo carmine. J. Clin. Ultrasound.

[100] Jeon H.J., Yoon J.S., Cho S.S., Kang K.O. (2012). Indigo carmine-induced hypotension in patients undergoing general anaesthesia. Singap. Med. J..

[101] Kennedy W.F., Wirjoatmadja K., Akamatsu T.J., Bonica J.J. (1968). Cardiovascular and respiratory effects of indigo carmine. J. Urol..

[102] Jeffords D.L., Lance P.H., DeWolf W.C. (1977). Severe hypertensive reaction to indigo carmine. Urology.

[103] Chu J.N., Lazar J., Badger J. (2015). A postoperative blue rash: Indigo carmine dye extravasation. Int. J. Dermatol..

[104] Isosu T., Satoh T., Oishi R., Imaizumi T., Hakozaki T., Obara S., Ikegami Y., Kurosawa S., Murakawa M. (2016). Effects of indigo carmine intravenous injection on noninvasive and continuous total hemoglobin measurement. J. Clin. Monit. Comput..

[105] Zippelius T., Hoburg A., Preininger B., Vörös P., Perka C., Matziolis G., Röhner E. (2013). Effect of indigo carmine on human chondrocytes in vitro. Open Orthop. J..

[106] Choulis N.H., Ray S.D. (2014). Miscellaneous drugs, materials, medical devices and techniques. Side Effects of Drugs Annual.

[107] Tsou M.P.M. (2003). Posterolateral percutane endoscopic lumbar discectomy. Oper. Technol. Orthop..

[108] Yao J., Trindade M.C.D. (2011). Color-aided visualization of dorsal wrist ganglion stalks aids in complete arthroscopic excision. Arthroscopy.

[109] Sheynkin Y.R., Starr C., Li P.S., Goldstein M. (1999). Effect of methylene blue, indigo carmine, and Renografin on human sperm motility. Urology.

[110] Yang J., Monk T.G., White P.F. (1991). Acute hemodynamic effects of indigo carmine in the presence of compromised cardiac function. J. Clin. Anesth..

[111] Yanagidate F., Hamaya Y., Dohi S. (2001). Vaginal indigo carmine-induced severe hypotension. Anesth. Analg..

[112] Luketic L., Murji A. (2016). Options to evaluate ureter patency at cystoscopy in a world without indigo carmine. J. Minim. Invasive Gynecol..

[113] Mahadevan M.M., Weitzman G.A., Hogan S., Breckinridge S., Miller M.M. (1993). Methylene blue but not indigo carmine is toxic to human luteal cells in vitro. Reprod. Toxicol..

[114] Imai K., Chikazawa K., Yonemori E., Kuwata T. (2022). Visualizing the dissectable layer for vaginal hysterectomy with indigo carmine. Eur. J. Obstet. Gynecol. Reprod. Biol..

[115] Higashimori A., Takahara M., Utsunomiya M., Fukunaga M., Kawasaki D., Mori S., Takimura H., Hirano K., Tsubakimoto Y., Nakama T. (2019). Utility of indigo carmine angiography in patients with critical limb ischemia: Prospective multi-center intervention study (DIESEL-study). Catheter. Cardiovasc. Interv..

[116] Monson F.C., Wein A.J., McKenna B.A., Whitmore K., Levin R.M. (1991). Indigo carmine as a quantitative indicator of urothelial integrity. J. Urol..

[117] Mimura M., Alameddine R.M., Korn B.S., Kikkawa D.O., Oku H., Sato B., Ikeda T. (2020). Endoscopic evaluation of lacrimal mucosa with indigo carmine stain. Ophthalm. Plast. Reconstr. Surg..

[118] Kubo K., Kimura N., Kato M. (2022). Texture and color enhancement imaging with indigo carmine dye accentuates slightly depressed early gastric cancer lesions. Clin. Gastroenterol. Hepatol..

[119] Yasuda T., Yagi N., Omatsu T., Hayashi S., Nakahata Y., Yasuda Y., Obora A., Kojima T., Naito Y., Itoh Y. (2021). Benefits of linked color imaging for recognition of early differentiated-type gastric cancer: In comparison with indigo carmine contrast method and blue laser imaging. Surg. Endosc..

[120] Resindra A., Monno Y., Imahori K., Okutomi M., Suzuki S., Gotoda T., Miki K. (2019). 3D reconstruction of whole stomach from endoscope video using structure-from-motion. Annu. Int. Conf. IEEE Eng. Med. Biol. Soc..

[121] González-Bernardo O.S., Riestra S., Vivas S., de Francisco R., Pérez-Martínez I., Castoño-García A., Jiménez-Beltrán V., Rollé V., Suárez P., Suárez A. (2021). Chromoendoscopy with indigo carmine vs virtual chromoendoscopy (iSCAN1) for neoplasia screening in patients with inflammatory bowel disease: A prospective randomized study. Inflamm. Bowel Dis..

[122] Lord R., Burr N.E., Mohammed N., Subramanian V. (2018). Colonic lesion characterization in inflammatory bowel disease: A systematic review and meta-analysis. World J. Gastroenterol..

[123] Fu K., Sano Y., Kato S., Fujii T., Nagashima F., Yoshino T., Okuna T., Yoshida S., Fujimori T. (2004). Chromoendoscopy using indigo carmine dye spraying with magnifying observation is the most reliable method for differential diagnosis between non-neoplastic and neoplastic colorectal lesions: A prospective study. Endoscopy.

[124] Kanamori T., Itoh M., Yoshimi N. (2002). Pressure dye-spray: A simple and reliable method for differentiating adenomas from hyperplastic polyps in the colon. Gastrointest. Endosc..

[125] Hurlstone D.P., Brown S., Cross S.S. (2003). The role of flat and depressed colorectal lesions in colorectal carcinogenesis: New insights from clinicopathological findings in high-magnification chromoscopic colonoscopy. Histopathology.

[126] Kiesslich R., Jung M., DiSario J.A., Galle P.R., Neurath M.F. (2004). Perspectives of chromo and magnifying endoscopy: How, how much, when, and whom should we stain?. J. Clin. Gastroenterol..

[127] Kudo S., Tamura S., Nakajima T., Yamano H., Kusaka H., Watanabe H. (1996). Diagnosis of colorectal timorous lesions by magnifying endoscopy. Gastrointest. Endosc..

[128] Buchner A.M. (2017). The role of chromoendoscopy in evaluating colorectal dysplasia. Gastroenterol. Hepatol..

[129] Pham N.B., Vu K.T., Nguyen N.H., Doan H.T., Tran T.T. (2022). Magnifying chromoendoscopy with flexible spectral imaging color enhancement, indigo carmine, and crystal violet in predicting the histopathology of colorectal polyps: Diagnostic value in a scare-setting resource. Gastroenterol. Res. Pract..

[130] Ng T.Y., Datta T.D., Kirimli B.I. (1976). Reaction to indigo carmine. J. Urol..

[131] Kawaguchi Y., Hashimoto H., Kitayama M., Hirota K. (2007). Intravenous indigo carmine might cause cerebral ischemia. Acta Anaesthesiol. Scand..

[132] Craik J.D., Khan D., Afifi R. (2009). The safety of intravenous indigo carmine to assess ureteric patency during transvaginal uterosacral suspension of the vaginal vault. J. Pelvic Med. Surg..

[133] Sutton E., Pietrzak A. (2016). Indigo carmine-induced hypotension in a parturient with idiopathic pulmonary artery hypertension, hypertrophic cardiomyopathy, and LAD myocardial bridging. J. Cardiothorac. Vasc. Anesth..

[134] O’Hara J.F., Connors D.F., Sprung J., Ballard L.A. (1996). Upper extremity discoloration caused by subcutaneous indigo carmine injection. Anesth. Analg..

[135] Choi J.W., Lee J.J., Kim G.H., Hong S.H. (2012). Extensive skin color change caused by extravasation of indigo carmine. Korean J. Anesthesiol..

[136] Porter J.F., McKay G., Choy K.H. (1999). The prediction of sorption from a binary mixture of acidic dyes using single and mixed isotherm variants of the ideal adsorbed solute theory. Chem. Eng. Sci..

[137] Kettle A.J., Clark B.M., Winterbourn C.C. (2004). Superoxide converts indigo carmine to isatin sulfonic acid: Implications for the hypothesis that neutrophils produce ozone. J. Biol. Chem..

[138] Savel J., Kosin P., Broz A. (2015). Indigo carmine degradation in the presence of maltose and ethanol. J. Inst. Brew..

[139] Benedict C.S., Bamforth C.W. (2016). Dissolved gases. Brewing Materials and Processes. A Practical Approach to Beer Excellence.

[140] Gaunt I.F., Kiss I.S., Grasso P., Gangolli S.D. (1969). Short-term toxicity study on indigo carmine in the pig. Food Cosmet. Toxicol..

[141] Hooson J., Gaunt I.F., Kiss I.S., Grasso P., Butterworth K.R. (1975). Long-term in toxicity carmine in mice. Food Cosmet. Toxicol..

[142] Hansen W.H., Fitzhugh O.G., Nelson A.A., Davis K.J. (1966). Chronic toxicity of two food colors, Brilliant Blue FCF and Indigotine. Toxicol. Appl. Pharmacol..

[143] Singh S., Das M., Khanna S.K. (1993). Bio-metabolism of green S and Indigo Carmine through caecal microflora of rats. Biochem. Biophys. Res. Commun..

[144] Ozaki A., Kitano M., Itoh N., Kuroda K., Furusawa N., Masuda T., Yamaguchi H. (1998). Mutagenicity and DNA-damaging activity of decomposed products of food colours under UV radiation. Food Chem. Toxicol..

[145] Dixit A., Goyal R.P. (2013). Evaluation of reproductive toxicity caused by indigo carmine on male swiss albino mice. Pharmacologyonline.

[146] Peng Y.J., Chen C.M., Li Y.F., Guo Y.T., Chen Y.T., Chao K.H., Yang J.J. (2023). Patent blue versus methylene blue and indigo carmine as a better dye for chromodiscography: In vitro staining efficacy and cytotoxicity study using bovine coccygeal intervertebral discs. Spine J..

[147] Yoo W.H., Kim S.R., Kim S.H., Lee J., Mok J., Shin D.H., Ahn H.Y., Eom J.S. (2023). Stability and safety of transbronchial dye mixture for preoperative localization in a porcine model. Thorac. Cancer.

